# Assessment of Bioflocculant Production by *Bacillus* sp. Gilbert, a Marine Bacterium Isolated from the Bottom Sediment of Algoa Bay

**DOI:** 10.3390/md9071232

**Published:** 2011-07-11

**Authors:** Piyo Nontembiso, Cosa Sekelwa, Mabinya V. Leonard, Okoh I. Anthony

**Affiliations:** Applied and Environmental Microbiology Research Group (AEMREG), Department of Biochemistry and Microbiology, University of Fort Hare, Private Bag X1314, Alice 5700, South Africa; E-Mails: pnosisi@yahoo.com (P.N.); sekco@webmail.co.za (C.S.); lmabinya@ufh.ac.za (M.V.L.)

**Keywords:** marine, Algoa bay, *Bacillus* sp. Gilbert, bioflocculant, polysaccharide

## Abstract

The bioflocculant-producing potentials of a marine bacteria isolated from the bottom sediment of Algoa Bay was investigated using standard methods. The 16S rDNA sequence analysis revealed 98% similarity to that of *Bacillus* sp. HXG-C1 and the nucleotide sequence was deposited in GenBank as *Bacillus* sp. Gilbert with accession number HQ537128. Bioflocculant was optimally produced when sucrose (72% flocculating activity) and ammonium chloride (91% flocculating activity) were used as sole sources of carbon and nitrogen, respectively; an initial pH 6.2 of the production medium; and Mg^2+^ as cation. Chemical analysis of the purified bioflocculant revealed the compound to be a polysaccharide.

## 1. Introduction

Bioflocculation is the dynamic process resulting from the synthesis of extracellular polymer by living cells [1]. Since flocculation in microbial systems was first reported by Louis Pasteur [2], bioflocculation has been investigated extensively and correlation was established between the accumulation of extracellular bioflocculants and cell aggregation [3]. Bioflocculants are essential polymers produced by microorganisms during their growth, with their flocculating activity being dependent on the characteristics of the flocculants. These have special advantages such as safety, strong effect, biodegradability and harmlessness to humans and the environment, which make them potentially suitable for application in drinking water and wastewater treatment, downstream processing, and fermentation processes [2].

There are three classes of flocculants, namely: (i) inorganic flocculants such as aluminum sulfate and polyaluminum chloride; (ii) organic synthetic flocculants such as polyacrylamide derivatives and polyethylene amine; and (iii) naturally occurring flocculants such as chitosan, sodium alginate, and microbial flocculants [4,5]. Inorganic and organic flocculanting agents such as those mentioned above are frequently used both in water treatment and fermentation industries because of their strong flocculating activity and low cost. However, studies have shown that synthetic flocculating substances may cause health and environmental problems. For example, the acrylamide monomer is not only a neurotoxin and a strong human carcinogen, but also non-degradable in nature [6]. On the other hand, bioflocculants produced by microorganisms are safe and biodegradable [7]. However, naturally occurring bioflocculants, although safe and biodegradable, show only weak flocculating activity in application [3]. Thus, the exploration for new biodegradable bioflocculants with strong flocculating activity is attracting wide research interest. Hence, in this paper, we assess the bioflocculant-producing potentials of a marine bacteria belonging to the *Bacillus* genus isolated from the bottom sediment of Algoa Bay in the Eastern Cape Province of South Africa.

Members of the *Bacillus* genus are Gram-positive, rod-shaped bacteria and a member of the division Firmicutes. *Bacillus* species can be obligate aerobes or facultative anaerobes, and they test positive for the enzyme catalase. They are ubiquitous in nature and include both free-living and pathogenic species [8]. They are also noted for efficient protein secretion system, as well as their ability to grow on several different and cheap carbon sources [9]. Also, the genus includes a variety of industrially important species with a history of safe use in both food and industry [10]. For example, species such as *Bacillus subtilis*, *Bacillus licheniformis*, *Bacillus megaterium*, *Bacillus amyloliquefaciens*, and *Bacillus clausii* offer several advantages in industrial applications [9] such as in agricultural biotechnology with several *Bacillus*-based products marketed as microbial pesticides, fungicides or fertilizers [11]. A member of this genus, *Bacillus amyloliquefaciens* is well known as the source of a natural antibiotic protein—barnase (a ribonuclease) and is also reported for much of the world production of α-amylase and protease [12]. In another study, Fujiwara *et al.* [13] purified a thermostable alkaline protease from a thermophilic alkaliphile, *Bacillus* sp. strain B18. *Bacillus* species have also been implicated in the production of biopolymers such as biosurfactants and exopolysaccharides which are mostly of great interest for microbially enhanced oil recovery. As example, *Bacillus licheniformis* JF-2 has been reported to produce the most effective biosurfactant—lychenisin [14]. In another case, the thermo- and halotolerant *Bacillus licheniformis* BAS50 was found to produce a powerful surface-active agent—lychenisin A [15,16]. Nevertheless, although a number of active compounds have been characterized from the genus *Bacillus*, however, reports regarding their implication in the production of bioflocculants are very limited.

## 2. Results and Discussion

**Figure 1:**
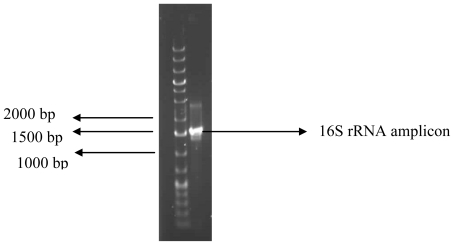
Polymerase Chain Reaction (PCR) product of the 16S rDNA of the test bacterium in 1% agarose gel.

Screening for bioflocculant production revealed our test bacteria to exhibit flocculating activity of 72.4%. Amplification of the 16S rRNA gene of the bacterium resulted in Polymerase Chain Reaction (PCR) product of expected size (approx. 1.5 kb) (Figure 1). BLAST (Basic Local Alignment Search Tool) analyses of the nucleotide sequence of the 16S rDNA revealed the bacterium to have 98% similarity to that of *Bacillus* sp. HXG-C1 and the nucleotide sequence was deposited in GenBank as *Bacillus* sp. Gilbert with accession number HQ537128. The colonies of the bacteria were light yellowish in color, rough, concentric, and of about 4 mm diameter when cultivated on M1 agar.

The geochemical characteristics of the sampling site water are shown in Table 1. The sediment samples were collected at a depth of approximately 7.25 m, and the temperature of the water at this depth was about 17.2 °C, while the conductivity was 46 mS/cm and the Salinity, pH, Turbidity, and Dissolved oxygen (DO) were 35.8 ppt, 8.42, 3.72 NTU and 7.12 mg/L respectively.

Bioflocculant production was affected by various factors, such as carbon and nitrogen sources, cations and initial pH of the production medium [1,5,17,18]. Optimization of these factors is essential since productivity and distribution of bioflocculant is dependent on the culture conditions [2]. The effects of the mentioned factors on the bioflocculant production by the test bacterium are shown in Table 2. Sucrose was found to be the best carbon source with a flocculating activity of 72.4%, while ammonium chloride was the best nitrogen source (91% flocculating activity). In addition, magnesium chloride appeared to be the best cation for bioflocculant production by the bacterium (72% flocculating activity).

The importance of carbon and nitrogen sources has been greatly emphasized and well documented for bioflocculant production [5]. However, studies have revealed that different microorganisms differ in requirement of such factors. For example, *Serratia ficaria* favoured lactose as the best carbon source for the production of the bioflocculant [19]. In the case of *Rhodococcus erythropolis*, glucose and fructose boosted the elongation of cells and flocculant production [20]. Also, in our previous report [18] *Virgibacillus* sp. Rob was observed to produce bioflocculant optimally in the presence of glucose and peptone as sole sources of carbon and nitrogen respectively. In this study, our test bacterium *Bacillus* sp. Gilbert preferred sucrose and ammonium chloride as carbon and nitrogen sources, respectively. A similar phenomenon where sucrose was utilized as the most favorable carbon source for the production of a bioflocculant produced by *Bacillus* sp. F19 was reported elsewhere [21]. Also, in another report [22], *Bacillus licheniformis* X14 preferred sucrose, starch and ethanol as favorable carbon sources for the production of ZS-7 bioflocculant, while ammonium chloride was effectively utilized as a nitrogen source.

The addition of cations stimulates flocculating activity by neutralizing and stabilizing the residual negative charge of functional groups and thereby forming bridges between particles [23]. Their role is therefore to enhance initial adsorption of flocculants on suspended particles by decreasing the negative charge on both the polymer and the particle, thereby improving the process of bioflocculation [23]. In this particular study, our test bacterium was stimulated by the addition of Ca^2+^, Mg^2+^, K^+^ and Fe^2+^. However, Mg^2+^ was the most favorable, hence was utilized in this present study. Similarly, the flocculating ability of TJ-F1 bioflocculant produced by *Proteus mirabilis* TJ-1 was enhanced by cations like Ca^2+^, Mg^2+^ and Fe^3+^ with Mg^2+^ being the best cation for that particular study [5]. In contrast to these findings, Mg^2+^ and K^+^ had a null effect on the production and activity of bioflocculant produced by *Virgibacillus* sp. Rob. However, that particular bioflocculant production and activity was stimulated by the presence of Fe^2+^ and Ca^2+^ [18].

The addition of salts increases the ionic strength of the kaolin suspension, decreasing the electrostatic forces. The effect on ionic strength increases with the charge and molar concentration of the ions. The increase on the ionic strength due to each salt amounted to 9.6 mM for MgCl_2_; 8.2 mM for CaCl_2_; 7.9 mM for FeSO_4_; and 4.1 mM for KCl. Therefore, the effect of CaCl_2_ and FeSO_4_ should be very similar, but lower than the effect of MgCl_2_, while that of KCl is expected to be least. However, experimental results show that replacing the CaCl_2_ by any of the other salts increased the flocculating activity. Therefore, the reduction of electrostatic forces was not the only explanation for the effect of salts on flocculating activity. Divalent ions can adsorb on anionic surface of the kaolin clay particles and act as anchoring points for the biofloculant chains, which increases its flocculating ability. However, they could also interact with anionic charged groups of different parts of the polysaccharide chain, making it less extended, and this could reduce its ability to form bridges. Hence, these two effects are less likely to be observed with KCl.

**Figure 2:**
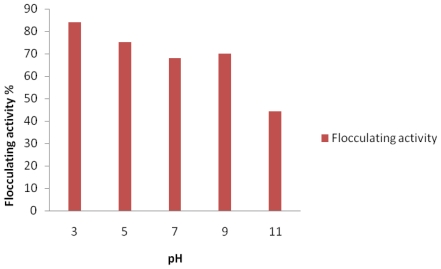
Effect of initial pH on bioflocculant activity by *Bacillus* sp. Gilbert.

The initial pH of the production medium is one of the factors affecting the production and flocculating activity of the bioflocculant [5,18,24]. Similarly, in this case, the bioflocculant production and activity of the studied bacteria strain was affected by the initial pH of the production medium, thus affecting flocculating activity. As shown in Figure 2, our test bacteria produced bioflocculant optimally at acidic initial pH of 3.0 (84% flocculating activity), and the flocculating activity decreased steadily as the pH tended towards alkalinity. It would appear that pH of the natural habitat of the test bacteria has no bearing on its bioflocculant production potential as the habitat had an alkaline pH of about 8.42.

Also, change of pH might vary the charge status of the bioflocculant and surface characteristics of suspended materials, hence resulting in variation of flocculating activity. In this case, the bioflocculant produced was found to be polysaccharide and, being produced at acidic pH, the H^+^ and COO^−^ of the polysaccharide is not expected to dissociate but rather hold together (COOH^+^), and consequently bond conveniently with the anionic particles of the kaolin clay suspension, resulting in enhanced flocculation.

Initial pH of culture media have been shown to variously affect the production of bioflocculants by different organisms. Yim *et al.* [25] reported bioflocculant produced by *Gyrodium impudicum* KG03 to have maximum activity at acidic pH 4.0. In another study [26], bioflocculant production and activity were greatly stimulated at alkaline pH 7.5. The bioflocculant produced by *Rhodococcus erythropolis* was reported to be active at neutral pH [4], while a recently reported bioflocculant produced by *Virgibacillus* sp. Rob preferred alkaline conditions [18].

**Figure 3:**
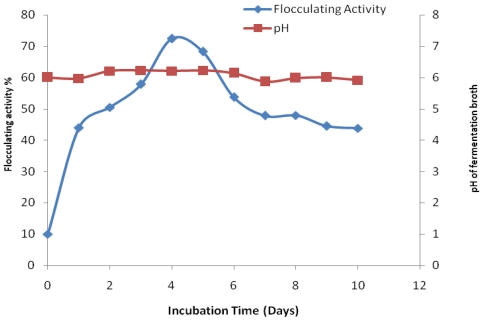
Time course of bioflocculant production by *Bacillus* sp. Gilbert.

Figure 3 shows time course of bioflocculant production by *Bacillus* sp. Gilbert in a production medium with initial pH 6.2 over a period of 10 days cultivation. The flocculating activity increased rapidly with increasing incubation time and reached a peak activity of 72.4% after four days of fermentation. Thereafter, the flocculating activity decreased steadily with the increase of culture time. The consequent decrease of flocculating activity may possibly be as a result of cell autolysis and enzymatic activity [19]. The pH of the medium was observed to be at constant within the first 2 days of cultivation after which it slightly increased and remained more or less constant throughout the cultivation period.

Microorganisms have been observed to differ in respect of culture times required for the production of their bioflocculants. Our test bacteria produced bioflocculant maximally at the end of the fourth day of fermentation suggesting that the bioflocculant was produced by biosynthesis. A similar phenomenon based on Shimforuya *et al.* [27] was observed whereby *Streptomycetes griseus* produced a bioflocculant with flocculating activity increasing with the increase of cultivation time. The maximum flocculating activity was also reached after four days and then decreased linearly with cultivation time. Based on a recent study [18], *Virgibacillus* sp. Rob was reported to also produce bioflocculant with maxima flocculating activity within the fourth day of cultivation. Deng *et al.* [11] reported bioflocculant produced by *Aspargillus parasiticus* to attain the highest flocculating activity in 96 h. However, in contrast to these findings, bioflocculant produced by *Serratia ficaria* reached its maximum flocculating activity on the third day of cultivation [19] whilst in case of *Citrobacter* sp. TKF04 bioflocculant exhibited maxima flocculating activity within one day [28].

A number of bioflocculants have been documented and the majority have been found to contain major components such as polysaccharides, proteins, lipids, glycolipids and glycoproteins [29,30]. The bioflocculant produced by our test bacterium was found to be mainly polysaccharide with the total sugar concentration of 22.5 mg/mL, and no protein was detected. Similar results were obtained with *Bacillus* sp. 450 [31], *Bacillus subtilis* IFO3335 [32], *Serratia ficaria* [19] and *Virgibacillus* sp. Rob [18] whereby the polymers were deduced to be mainly polysaccharides.

## 3. Experimental Section

### 3.1. Source of Bacteria and Culture Media

Several marine bacteria previously isolated from the bottom sediments of Algoa Bay in the Eastern Cape of South Africa as part of the culture collections of the Applied and Environmental Microbiology Research Group (AEMREG), University of Fort Hare, Alice, South Africa were screened for bioflocculant production. The composition of the production medium is as described by previous authors [18,24] and includes the following: 10 g glucose, 1 g peptone, 0.3 g MgSO_4_·7H_2_O, 5 g K_2_HPO_4_, 2 g KH_2_PO_4_ in 1 L filtered sterilized seawater; and the initial pH of the medium was adjusted to 7.0 with NaOH (0.1 M) and HCl (0.1 M). All media solutions were sterilized by autoclaving.

### 3.2. Screening for Flocculant Producing Microorganisms

Each bacterial isolate was inoculated into a McCartney bottle containing 5 mL of production medium and incubated on a shaker at 160 rpm for 3 to 5 days at 28 °C. At the end of the incubation period, the fermentation broth was centrifuged at 4000 g for 10 min to separate the cells, and the cell free culture supernatant was assayed for flocculating activity.

### 3.3. Measurement of Flocculating Activity

Using a suspension of kaolin clay as test material, flocculating activity was measured according to the method of Kurane *et al.* [4] as modified by Zhang *et al.* [24]. Three milliliters of 1% CaCl_2_ and 2.0 mL of the cell-free supernatant were added into 95 mL of kaolin suspension (4.0 g/L) in 250 mL flask. The mixture was vigorously stirred and allowed to stand for 5 min at room temperature. The optical density (OD_550nm_) of the clarifying solution was measured with a ThermoSpectronic spectrophotometer (Helios Epsilon, USA) at 550 nm. A control experiment was prepared in the same way but the cell-free supernatant was replaced with the un-inoculated production medium. The flocculating activity was estimated from the formula:

Flocculating activity={(A-B)/A}×100%

where A and B were optical densities of the control and samples respectively at 550 nm.

### 3.4. Effects of Culture Conditions on Bioflocculant Production

Effect of culture conditions such as carbon and nitrogen sources, cations and initial pH were assessed as described elsewhere [18,19,26]. Carbon source candidates included glucose, sucrose, fructose and starch, while the nitrogen source candidates included ammonium sulphate and ammonium chloride (as inorganic nitrogen sources), as well as urea and peptone (as organic nitrogen sources). The metal ions candidates included CaCl_2_, KCl, MgCl_2_ and FeSO_4_, while the effect of pH was evaluated by varying the initial pH of the culture media using HCl (0.1 M) and NaOH (0.1 M) in the pH range of 3–12 [25]. In the experiments on the effects of medium composition, only the carbon or nitrogen sources were replaced while the other constituents, temperature (28 °C) and shaking speed (160 rpm) were kept constant.

### 3.5. Time Course Experiment

For the time course experiment, the composition of the medium for the bioflocculant production was as follows: 10 g of sucrose, 1.0 g of ammonium chloride, 0.3 g of MgSO_4_·7H_2_O, 5 g of K_2_HPO_4_ and 0.2 g of KH_2_PO_4_ in 1 L of filtered natural sea water [24]. The isolate was cultured under optimal growth conditions. Fifty millilitres of saline solution was inoculated with a loop full of pure colonies of the isolate, vortexed and the suspension standardized to OD_660nm_ 0.1.

Time course assays were thereafter conducted in accordance with our previous description [18]. Briefly, the standardized saline solution was used as seed culture for inoculum preparation. Seed culture (1% v/v) was inoculated into 150 mL of medium in 500 mL flasks (prepared in duplicates) and incubated on a rotatory shaker (160 rpm) at 28 °C. Samples were drawn every 24 h for a period of 10 days. Two milliliters of culture broth was centrifuged at 4000 g for 10 min and the cell free supernatant was used to determine the flocculating activity. The pH of the broth samples was also measured.

### 3.6. Bioflocculant Purification and Analyses

To purify the bioflocculant, the methods described by Chang *et al.* [33] and Chen *et al.* [34] were followed. Briefly, the fermentation broth was centrifuged at 8000 g for 30 min to remove the bacterial cells and one volume of distilled water was added to the supernatant and centrifuged at 8000 g for 15 min to remove insoluble substances. The supernatant was then mixed with two volumes of ethanol, stirred and left standing at 4 °C for 12 h, after which the supernatant was decanted and the precipitate vacuum-dried to obtain crude biopolymer. The crude product was dissolved in distilled water and then mixed with one volume of chloroform/*n*-butyl alcohol (5:2, v/v). After stirring, the mixture was left standing at room temperature (about 20 °C) for 12 h. The upper phase was separated, centrifuged at 3000 g for 15 min and the supernatant concentrated at 40 °C. Two volumes of ethanol were added, the precipitate recovered, vacuum-dried and then re-dissolved in distilled water to obtain a purified bioflocculant. The total sugar content of the bioflocculant was determined using the Phenol-sulfuric acid method with glucose as the standard solution. The protein content was measured by Folin-Lowry method with bovine serum albumin (BSA) as the standard as described by Lachhwani [26].

### 3.7. Identification of the Bioflocculant-Producing Bacterium

The bacterium was identified using molecular technique based on the 16S rRNA gene amplification by polymerase chain reaction (PCR) followed by sequencing of the amplified gene. Template DNA of the bacterium for use in the PCR was prepared using the boiling method as described elsewhere [35]. The PCR amplification reaction was carried out following our previous description [18] in 50 μL reaction volume containing 2 mM MgCl_2_, 2 U Supertherm Taq polymerase, 150 mM of each dNTP, 0.5 mM of each primer (F1: 59-AGAGTTTGATCITGGCTCAG-39; I = inosine and primer R5: 59-ACGGITACCTTGTTACGACTT-39) and 2 μL template DNA. Primer F1 and R5 binds to base positions 7–26 and 1496–1476 of the 16S rRNA gene of *Streptomyces ambofaciens* ATCC 23877, respectively [35]. The primers in this study were used to amplify nearly full-length 16S rDNA sequences. The PCR programme used was an initial denaturation (96 °C for 2 min), 30 cycles of denaturation (96 °C for 45 s), annealing (56 °C for 30 s) and extension (72 °C for 2 min), and a final extension (72 °C for 5 min). Gel electrophoresis of PCR products were conducted on 1% agarose gels to confirm that a fragment of the correct size had been amplified.

## 4. Conclusions

Our test bacterium, *Bacillus* sp. Gilbert appears to be a promising source of polysaccharide bioflocculant(s). Optimal conditions for its bioflocculant production included sucrose and ammonium chloride as carbon and nitrogen sources respectively, with acidic pH 3.0 and Mg^2+^ as cation. It is anticipated that *Bacillus* sp. Gilbert has tremendous potential and may be an attractive candidate for use in water treatment and other relevant biotechnology applications. Further characterization and development of process conditions for large-scale production and practical application of the purified bioflocculant are subjects of on-going investigation in our group.

## Figures and Tables

**Table 1:** Geochemical properties of the sampling site water.

Depth (m)	Temperature (°C)	Conductivity (mS/cm)	Salinity (ppt)	pH	Turbidity (NTU)	DO (mg/L)
7.25 ± 0.19	17.2 ± 0.03	46 ± 0.03	35.8 ± 0.04	8.42 ± 0.26	3.72 ± 1.05	7.12 ± 0.5

DO: Dissolved oxygen; ppt: Parts per thousand.

**Table 2:** Effects of composition medium on the bioflocculant production and activity.

**Carbon source**	**Glucose**	**Sucrose**	**Fructose**	**Starch**
Flocculating activity (%)	65	72.4	59	–

**Nitrogen source**	**Peptone**	**Ammonium sulphate**	**Urea**	**Ammonium chloride**
Flocculating activity (%)	65	–	56	91

**Cations**	**Calcium chloride**	**Magnesium chloride**	**Iron sulphate**	**Potassium chloride**
Flocculating activity (%)	65	72	70	69

Note: (–) denotes no flocculating activity.
